# Colossal Permittivity Characteristics of (Nb, Si) Co-Doped TiO_2_ Ceramics

**DOI:** 10.3390/ma15134701

**Published:** 2022-07-05

**Authors:** Hicham Mahfoz Kotb, Adil Alshoaibi, Javed Mazher, Nagih M. Shaalan, Mohamad M. Ahmad

**Affiliations:** 1 Department of Physics, College of Science, King Faisal University, P.O. Box 400, Al-Ahsa 31982, Saudi Arabia; adshoaibi@kfu.edu.sa (A.A.); jkhan@kfu.edu.sa (J.M.); nmohammed@kfu.edu.sa (N.M.S.); mmohamad@kfu.edu.sa (M.M.A.); 2 Physics Department, Faculty of Science, Assiut University, Assiut 71516, Egypt; 3 Department of Physics, Faculty of Science, The New Valley University, El-Kharga 72511, Egypt

**Keywords:** ceramics, sintering, relative permittivity, relaxation

## Abstract

(Nb^5+^, Si^4+^) co-doped TiO_2_ (NSTO) ceramics with the compositions (Nb_0.5_Si_0.5_)_x_Ti_1−x_O_2_, x = 0, 0.025, 0.050 and 0.1 were prepared with a solid-state reaction technique. X-ray diffraction (XRD) patterns and Raman spectra confirmed that the tetragonal rutile is the main phase in all the ceramics. Additionally, XRD revealed the presence of a secondary phase of SiO_2_ in the co-doped ceramics. Impedance spectroscopy analysis showed two contributions, which correspond to the responses of grain and grain-boundary. All the (Nb, Si) co-doped TiO_2_ showed improved dielectric performance in the high frequency range (>10^3^ Hz). The sample (Nb_0.5_Si_0.5_)_0.025_Ti_0.975_O_2_ showed the best dielectric performance in terms of higher relative permittivity (5.5 × 10^4^) and lower dielectric loss (0.18), at 10 kHz and 300 K, compared to pure TiO_2_ (1.1 × 10^3^, 0.34). The colossal permittivity of NSTO ceramics is attributed to an internal barrier layer capacitance (IBLC) effect, formed by insulating grain-boundaries and semiconductor grains in the ceramics.

## 1. Introduction

Titanium dioxide (TiO_2_) is an interesting inorganic material used in several important applications such as photocatalysis, gas sensing and medical applications, due to its chemical stability at room temperature and its wide band gap [1]. The room temperature relative permittivity (ε′) of pure TiO_2_ is ~100, which is not suitable for practical energy storage applications. Interestingly, recent studies reported colossal relative permittivity (CP) for co-doped TiO_2_ in the form (A, B)_x_Ti_1−x_O_2_, where A is pentavalent and B is tri- or bivalent cations [2,3,4,5,6]. The origin of CP of the co-doped TiO_2_ ceramics is controversially discussed in the literature. Several models were proposed to explain the colossal relative permittivity and low dielectric loss (tanδ = ε″/ε′, where ε′ and ε″ are the real and imaginary part of the complex relative permittivity) of co-doped TiO_2_. It seems there is no universal model that can be applied for all cases. For example, the electron-pinned defect dipole (EPDD) model was successful for (Nb^5+^, In^3+^) co-doped TiO_2_ [7,8,9,10]. This model suggests that the electrons produced by the donors (Nb^5+^) would reduce Ti^4+^ to Ti^3^. In these conditions, defect complexes such as the triangular shaped ${\mathrm{In}}_{2}^{3+}V_{O}^{••}{\mathrm{Ti}}^{3+}$ are created in the rutile structure of TiO_2_ and are able to inhibit the free electrons from long-range motion. Therefore, CP and low tanδ were expected for these co-doped TiO_2_. In addition to the EPDD model, the internal barrier layer capacitance (IBLC) model [11,12] has also been suggested as a possible reason for the colossal permittivity of (donor, acceptor) co-doped TiO_2_ [2,13]. The IBLC model is widely accepted for the interpretation of CP of electrically heterogeneous ceramics. In IBLC, conducting grains are separated by the electrically insulating grain-boundaries. The applied alternating voltage on the sample displaces the charge carriers from the semiconductor grains where they pile up at the thin resistive grain boundaries. The capacitors, thus formed, lead to a colossal effective relative permittivity (ε′) of the ceramic. Considering the IBLC model, the use of acceptors is not compulsory in order to obtain CP in co-doped TiO_2_. For example, Yang et al. reported on the colossal permittivity in (Nb^5+^, Zr^4+^) co-doped TiO_2_ [14]. Doping of CCTO with SiO_2_ has been reported as promoting the dielectric properties of CCTO, due to abnormal grain growth [15]. Doping of NiO with Si^4+^ was effective in increasing the grain boundary resistance due to the formation of a Si-rich secondary phase at the grain-boundaries, which resulted in decreasing the dielectric loss in co-doped NiO ceramics [16]. The phase purity of co-doped TiO_2_ is dependent on the solubility of dopants in TiO_2_, which can be predicted by the Hume–Rothery rule [14,17]. According to this rule, a substitutional-solid solution is more likely to form if the difference between the ionic radius of the doping element and the matrix element is less than 15%. In cases where an ionic radius mismatch is greater than 15%, the formation of an interstitial solid solution is more favorable. Most studies opted to choose dopants of ionic radii close to the Ti^4+^ to avoid the formation of a secondary phase in the prepared ceramics. Nevertheless, the effect of a secondary phase on the dielectric properties of ceramics with a targeted crystalline phase is not easy to predict in most cases [18,19]. The smaller ionic radius of Si^4+^ (radius = 40 pm) compared to Ti^4+^ (radius = 60.5 pm) would result in a secondary phase in the resulting ceramics. In the present work, we investigated the dielectric and structural properties of (Nb_0.5_Si_0.5_)_x_Ti_1−x_O_2_ (x = 0, 0.025, 0.050 and 0.1) over a wide range of frequencies and temperatures. It was found that NSTO ceramics have improved dielectric performance compared to pure TiO_2_ while the sample (Nb_0.5_Si_0.5_)_0.025_Ti_0.975_O_2_ ceramic showed the best improved dielectric performance.

## 2. Experiment Procedure

### 2.1. Materials

Anatase TiO_2_ (99.8%, Aldrich, St. Louis, MI, USA), Nb_2_O_5_ (99.99%, Aldrich) and SiO_2_ (99.99%, Aldrich) were used for the synthesis of the powder of (Nb_0.5_Si_0.5_)_x_Ti_1−x_O_2_, x = 0, 0.025, 0.050 and 0.1. These samples will be referred to as NSTO.

### 2.2. Synthesis of (Nb_0.5_Si_0.5_)_x_Ti_1−x_O_2_ Ceramics

Stoichiometric amounts of the elemental oxides were weighed according to the molar ratio of (Nb_0.5_Si_0.5_)_x_Ti_1−x_O_2_, then milled in a Retsch PM400 machine for 20 h with a rotation speed of 200 rpm using pots and balls made of tungsten carbide. The obtained powder was pressed (320 MPa) into pellets, 10 mm in diameter and 2 mm in thickness. Subsequently, the pellets were sintered in air inside an electric tubular furnace at a temperature of 1500 °C for 10 h.

### 2.3. Characterization Methods

Field-emission scanning electron microscope (FE-SEM) (Joel, Tokyo, Japan, SM7600F) and powder X-ray diffraction (XRD) (Stoe and Cie GmbH, Darmstadt, Germany, Cu Kα1 radiation) techniques were used to study the morphology and phase purity of the prepared ceramics. XRD measurements were taken across the range 20° ≤ 2θ ≤ 80° with steps of 0.02°. Raman spectra were recorded with a fully integrated confocal Raman microscope (HORIBA Scientific, Piscataway, NJ, USA) in a back-scattering configuration. All the samples were excited by a He-Cd laser (wavelength 442 nm, 100 mW). The number of gratings in the Raman spectrometer was 1800 L/mm. Impedance spectroscopy (IS) measurements were conducted in dry a nitrogen atmosphere using the system turnkey concept 50 from Novocontrol in the temperature range 200–400 K over the frequency range 1 Hz–1 MHz at an oscillation voltage of 0.1 V. The temperature of the sample was automatically controlled by Quatro Cryosystem. Silver paint was applied to both sides of each pellet before the electrical measurements were taken.

## 3. Results and Discussion

**Figure 1:**
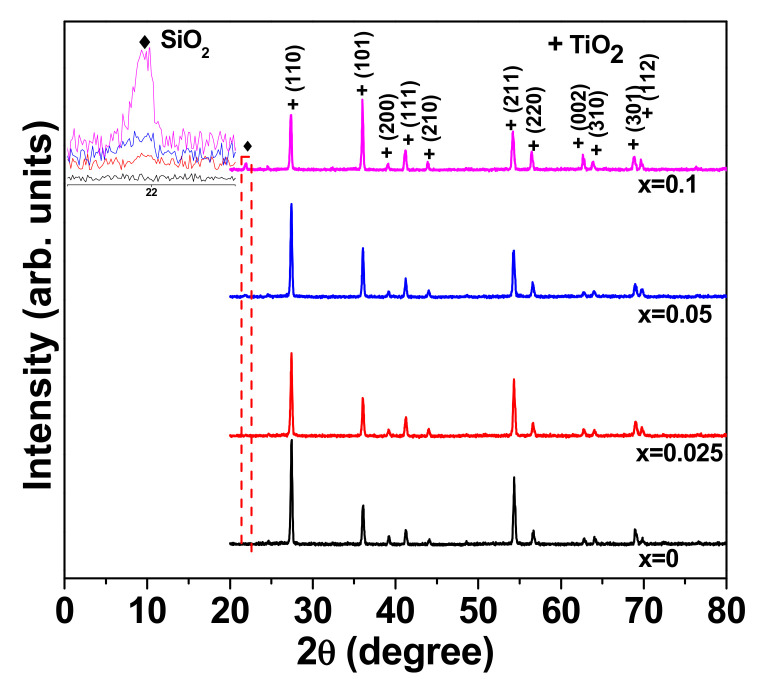
The XRD patterns of the sintered NSTO ceramics.

The XRD patterns of the sintered NSTO ceramics are shown in Figure 1. All the samples present the tetragonal rutile TiO_2_ (JCPDS 21-1276) as the main phase. The lattice parameters as well as the cell volume were calculated for each sample from the angle and hkl values of the main diffraction peaks using UNITCELL software. These values are summarized in Table 1. Compared to the un-doped TiO_2_, (Nb, Si) co-doped ceramics (x > 0) show higher values for lattice parameters and the cell volume. These results highlight the effect of the substitution of small Ti^4+^ ions (radius = 60.5 pm) by the larger Nb^5+^ ions (radius = 64 pm) in the TiO_2_ matrix. Moreover, a tiny impurity peak is observed in the XRD of doped samples at 21.82° and corresponds to SiO_2_ (JCPDS 47-718). Compared to the (110) peak of the main TiO_2_ rutile phase, the intensity of the impurity peak is considerably low, but increases with increasing the doping content.

**Figure 2:**
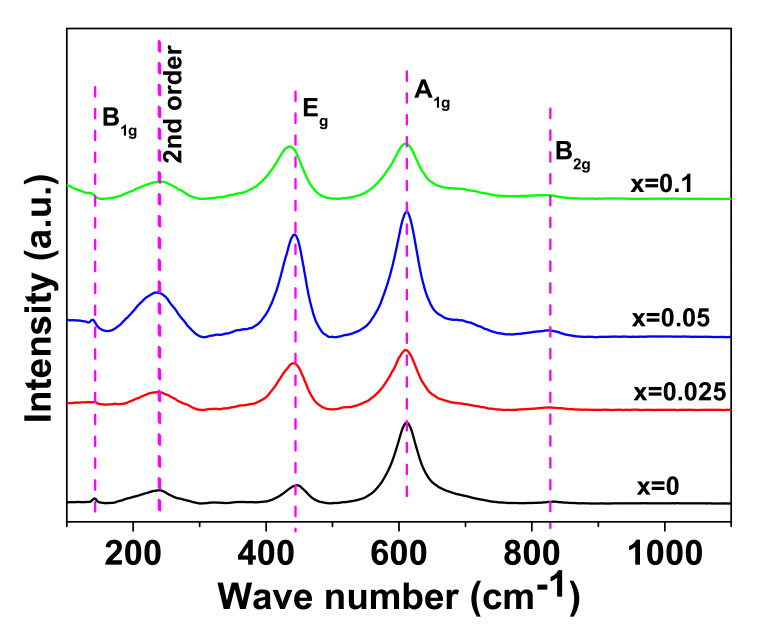
Raman spectra of the sintered ceramics of NSTO.

Raman spectroscopy is known for its usefulness in confirming the anatase or rutile phase of TiO_2_ [20]. Figure 2 represents the room temperature Raman spectroscopy over the range of 100–1100 cm^−1^ for NSTO ceramics. Four peaks are observed at ~143 cm^−1^, ~447 cm^−1^, ~612 cm^−1^ and ~826 cm^−1^. These peaks correspond to the typical first-order Raman-active modes of rutile TiO_2_: B_1g_, E_g_, A_1g_ and B_2g_, respectively [21]. In addition, a peak is observed at ~237 cm^−1^, which is a second-order scattering feature [21]. Therefore, Raman spectroscopy confirmed that all NSTO ceramics possess the rutile phase structure, which is in agreement with the XRD results. As seen in Figure 2, the E_g_ slightly shifted toward a lower wave number for the sample with x = 0.1, which might be due to the evolution of lattice distortion and the movement of oxygen along c-axis with the increase in dopants content [22].

**Figure 3:**
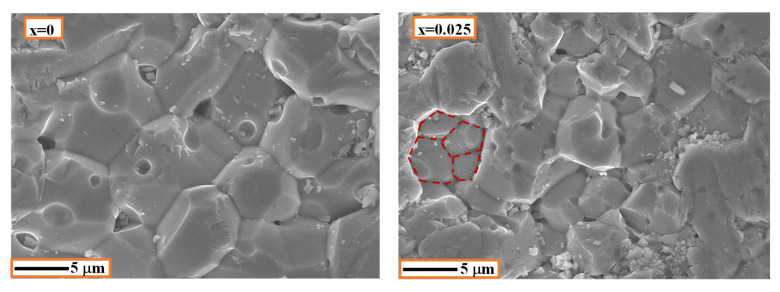

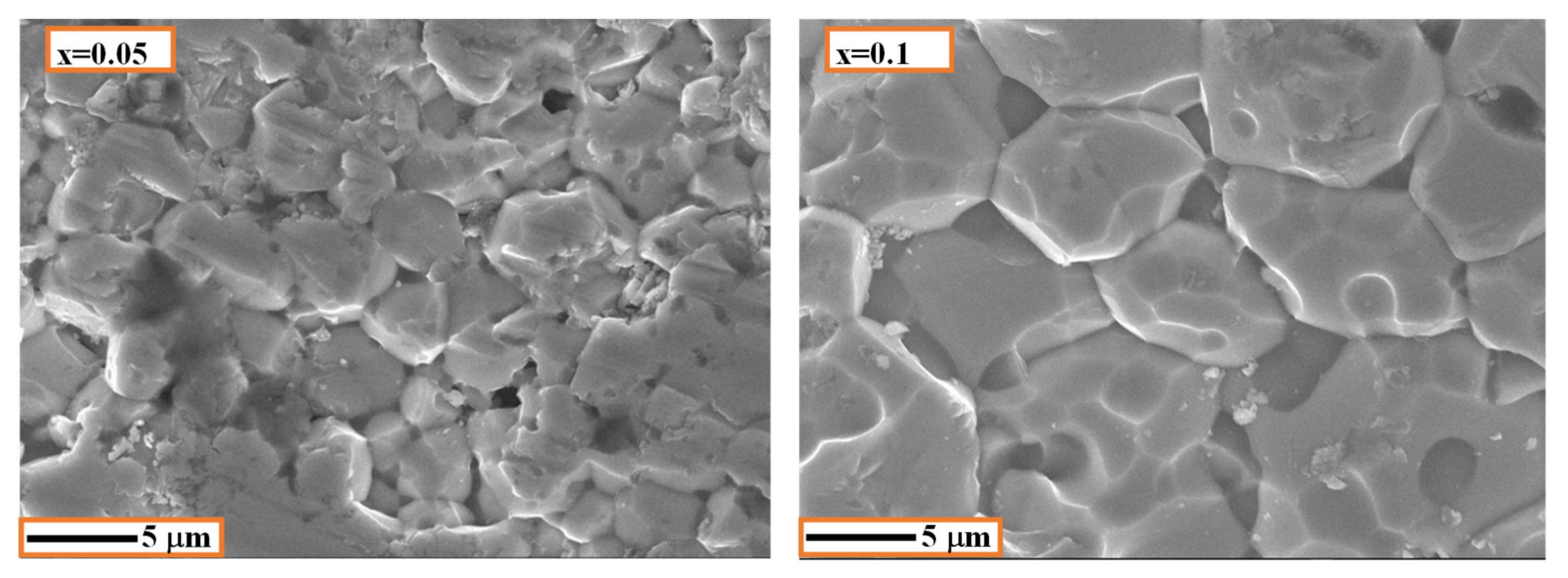
FE-SEM morphology of NSTO ceramics.

Figure 3 shows FE-SEM micrographs of the scratched surface of NSTO ceramics. The grains and grain-boundaries are clearly seen for all the samples. Using the lineal intercept method [23,24], the average grain size was found to be 9.1 ± 1.6, 5.6 ± 1.1, 4.8 ± 0.9 and 9.4 ± 1.6 µm for the samples with x = 0, 0.025, 0.05 and 0.1, respectively. Therefore, the average grain size tends to decrease with increasing the dopants content up to x = 0.05. Additionally, the grain in the co-doped samples seems to be composed of smaller sub-grains as shown by the red-dashed line in the SEM micrograph of the sample x = 0.025 (Figure 3). Similar observations on the effect of doping content on the grain size have been reported [25,26,27]. This behavior was attributed to the effect of the secondary phase in inhibiting grain growth [25] and/or the increased number of nuclei centers with increasing doping content [27].

**Figure 4:**
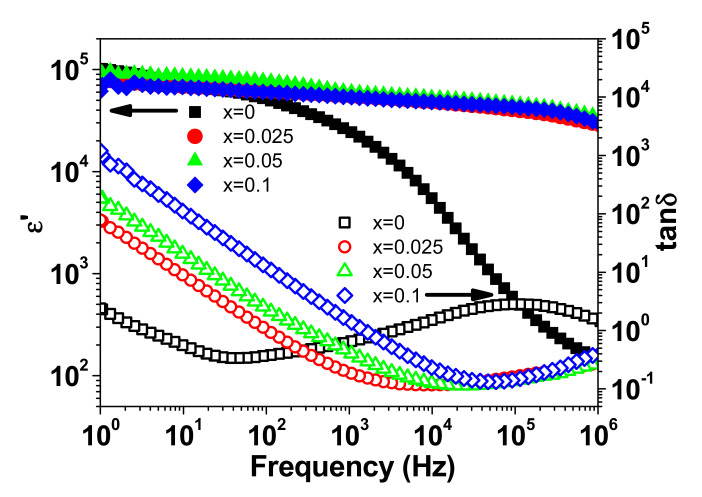
Frequency dependence of ε′ and tanδ at room temperature for NSTO ceramics.

Figure 4 shows the frequency dependency of relative permittivity (ε′) and dielectric loss (tanδ) of NSTO ceramics. It can be seen that (Nb, Si) co-doped TiO_2_ (x > 0) ceramics have improved dielectric properties in terms of higher ε′ and lower tanδ compared to the un-doped sample (x = 0). Among these ceramics, the sample with x = 0.025 showed the minimum tanδ of ~0.11 and colossal permittivity (ε′~4.8 × 10^4^) at 10 kHz and room temperature. This dielectric performance is close to that of (Nb, La) [25], (Ta, In) [6] and (Nb, Eu) [28] co-doped TiO_2_ but lower than (Ta, Mg) [29], (Ta, Y) [30] (Ta, Ho) [31] (Nb, Zr) [14] co-doped TiO_2_.

**Figure 5:**
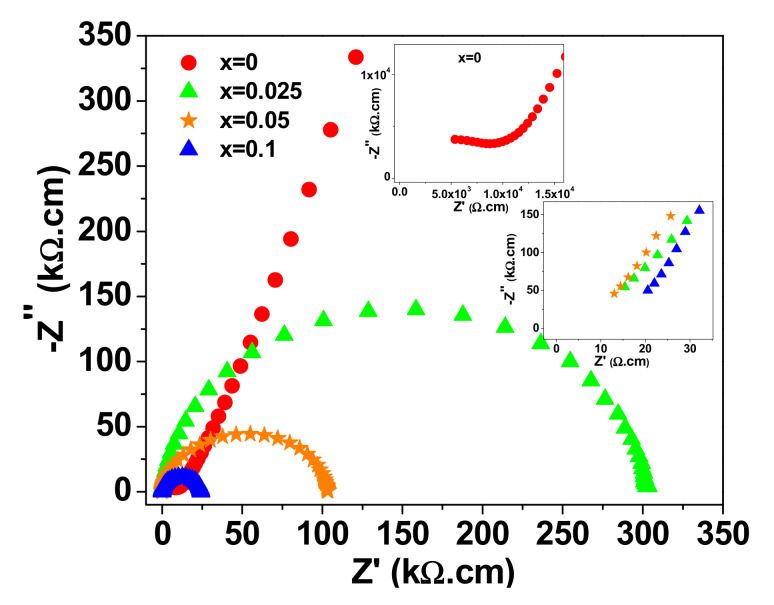
Impedance complex plane plot (Z*) at room temperature for NSTO ceramics.

Figure 5 depicts the room temperature complex impedance (Z* = Z′ + iZ″) plots for the four ceramic samples. The impedance spectrum of every sample is comprised of a large semicircular arc that covers most of the frequency range. However, a close examination at the high-frequency region reveals that the aforementioned arc does not pass through the origin. A second circular arc and non-zero intercept with the real Z′ axis is observed in the high frequency region for the samples with x = 0 and x > 0, respectively. This behavior is similar to what is reported for CaCu_3_Ti_4_O_12_ [32] and co-doped TiO_2_ [2,13]. The arc at high frequency (/non zero intercept) is attributed to the response of the semiconductor component (grains), while the larger arc represents the response of electrically insulating component (grain-boundaries). The resistances of grains (R_g_) and grain-boundaries (R_gb_) can be calculated from the intercepts of the corresponding arcs with the axis of the real part of the impedance (Z′) [11]. The room temperature values of R_g_ and R_gb_ are summarized in Table 2. It can be seen that the NSTO ceramics have an electrically heterogeneous structure of semiconducting grains and resistive grain-boundaries. It is observed that the change in R_gb_ with increasing doping content is more pronounced than in R_g_. This result indicates that (Nb^5+^, Si^4+^) co-doping affects, principally, the grain-boundary resistance of TiO_2_. Moreover, R_g_ and R_gb_ decrease gradually with increasing doping content because of the increase in the concentration of free carriers as a result of doping. The sintering temperature is reported to affect the dielectric performance of pure rutile TiO_2_ [18,33,34]. For example, ε′ was found to increase from 400 to 1.3 × 10^4^ with increasing the sintering temperature from 1200 to 1450 °C [33]. In our case, the undoped TiO_2_ has a performance comparable to that found in the literature for the rutile TiO_2_, which is sintered at high temperature [18]. In this reference, the dielectric constant and tanδ of pure TiO_2_ ceramics at 1 kHz and room temperature were found to be 10^3^ and ~0.4, respectively. As seen in Figure 4, the co-doped NSTO samples (x > 0) have comparatively higher relative permittivity (ε′) and lower dielectric loss (tanδ), in the frequency range > 10^3^ Hz, than the pure TiO_2_ (x = 0). As shown by the FE-SEM micrograph in Figure 3, the grain of the co-doped ceramic is composed of smaller grains. Therefore, we believe that the co-doping results in more grain-boundary barriers in front of the charge carriers, which causes the reduced tanδ values for co-doped ceramics in the high frequency range.

**Figure 6:**
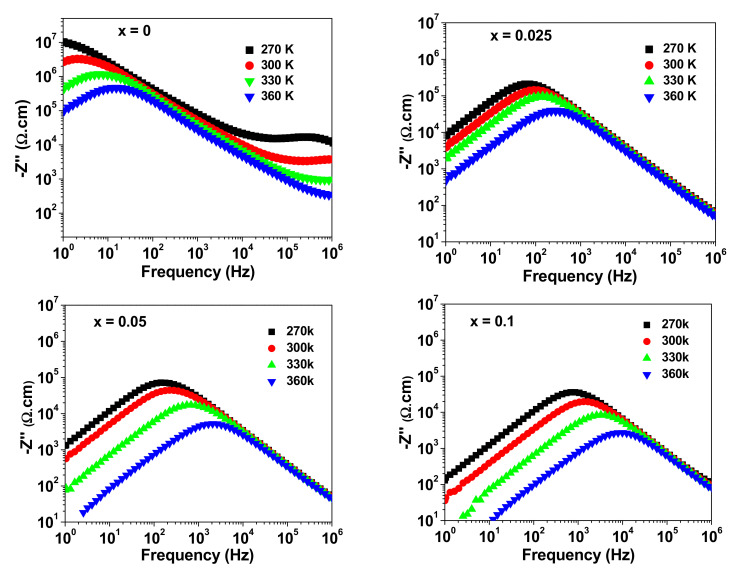
Frequency dependence of −Z″ at different temperatures for NSTO ceramics.

Figure 6 shows the variation in the imaginary part of the impedance (Z″) with frequency at selected temperatures for NSTO ceramics. At a given temperature, the spectrum of Z″ shows a peak with maximum value $Z_{\max}^{\prime \prime }$ at a frequency f_max_. With increasing temperature, $Z_{\max}^{\prime \prime }$ decreases while f_max_ shifts to higher frequencies. According to the inter barrier layer capacitance (IBLC), the dependence of the imaginary part of the impedance (Z″) on the angular frequency (ω) is given by [35,36]:

(1) $$-Z\prime \prime \left(\omega \right)=R_{g}\left[\frac{{\omega R}_{g}C_{g}}{1+{\left({\omega R}_{g}C_{g}\right)}^{2}}\right]+R_{g.b}\left[\frac{{\omega R}_{\mathrm{gb}}C_{\mathrm{gb}}}{1+{\left({\omega R}_{\mathrm{gb}}C_{\mathrm{gb}}\right)}^{2}}\right]$$

where R_g_ (/R_gb_) and C_g_ (/C_gb_) are the resistances and capacitances of the grain (/grain-boundaries), respectively. Consequently, for electrically heterogeneous materials with R_gb_ >> R_g_, the overall resistance of the sample R ≈ R_gb_ and can be calculated from $Z_{\max}^{\prime \prime }$ by the relationship:

(2) $$-Z_{\max}^{\prime \prime }=\frac{R_{\mathrm{gb}}}{2}$$

Moreover, the relaxation time (τ) is related to the frequency f_max_ by the relationship:

(3) $$\tau =\frac{1}{\omega }=\frac{1}{2{\pi f}_{\max}}$$

**Figure 7:**
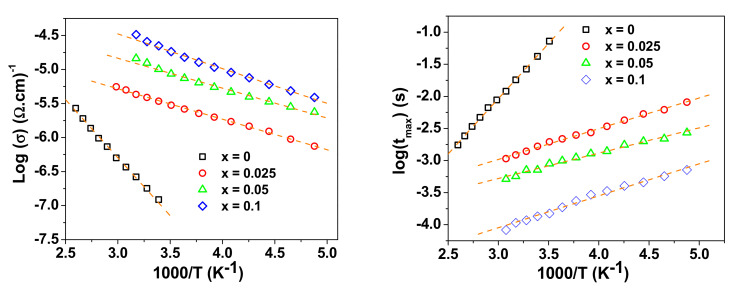
The Arrhenius plots of the conductivity σ (**left**) and relaxation time τ (**right**) in the grain-boundaries for NSTO ceramics. The dashed line represents the line of best fit.

The activation energy for conduction (E) and for relaxation (U) were calculated from the Arrhenius plots in Figure 7 using the relationships:

(4) $$\sigma =\sigma_{0}\;e^{-\left(\frac{E}{K_{B}T}\right)}$$

where σ_0_ is the pre-exponential factor, K_B_ is Boltzmann constant and T is absolute temperature.

(5) $$\tau =\;\tau_{0}\;e^{\left(\frac{U}{k_{B}T}\right)}$$

where τ_0_ is the pre-factor and U is the activation energy for the relaxation process.

The calculated values of activation energies from the spectra of M″ are given in Table 3. It can be seen that the co-doped NSTO samples (x > 0) have activation-energy values considerably lower than the pure TiO_2_ (x = 0), which correlate with the lower resistivity of these samples.

**Figure 8:**
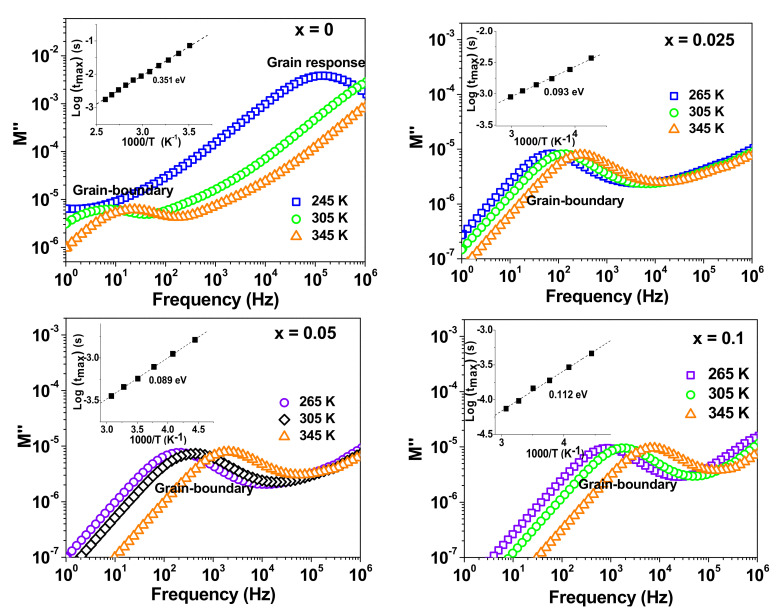
Frequency dependence of M′′ at different temperatures for NSTO ceramics.

Moreover, the modulus formalism has been deployed to further study the conductivity relaxation behavior of the NSTO ceramics. Figure 8 shows the spectra of the imaginary part M″ of the electric modulus M* = M′ + iM″ = 1/ε*, where ε* is the complex relative permittivity. At room temperature, the spectrum of M″ of the un-doped TiO_2_ (x = 0) shows two resolved peaks. For the co-doped samples (x > 0), only the low frequency peak is observed within the studied frequency range. The low and high frequency M″ peaks are attributed to the contribution of grain-boundary and grain, respectively. Furthermore, the observed peaks shift towards higher frequency with increasing temperature. The relaxation time τ is related to the peak frequency (f_max_) as τ = 1/2πf_max_. The insets of Figure 4 show the log(τ) versus 1000/T curves, which are fitted according to the Arrhenius equation. The calculated activation energy of the grain-boundary is 0.351 eV, 0.093 eV, 0.089 eV and 0.112 eV for the samples with x = 0, 0.025, 0.05 and 0.1, respectively. These values are comparable to the activation energies obtained from the spectra of Z″, as previously presented in Table 3.

The activation-energy values for the doped ceramics (x > 0) are comparable to the value of 0.1 eV reported for the dipolar relaxation due to electrons hopping between Ti^3+^ and Ti^4+^ ions in co-doped TiO_2_ [37]. Substituting of Nb^5+^ for Ti^4+^ resulting in a distorted lattice and the reduction of some Ti^4+^ to Ti^3+^. This process may be expressed using Kroger-Vink notations as follows [38,39]:

(6) $${\mathrm{Nb}}_{2}O_{5}+2{\mathrm{TiO}}_{2}=2{\mathrm{Nb}}_{\mathrm{Ti}}^{•}+2{\mathrm{Ti}}_{\mathrm{Ti}}^{\prime }+8O_{O}+\frac{1}{2O_{2}}$$

(7) $$2{\mathrm{Ti}}^{4+}+2e=2{\mathrm{Ti}}^{3+}$$

## 4. Conclusions

(Nb_0.5_Si_0.5_)_x_Ti_1−x_O_2_, (x = 0, 0.025, 0.050 and 0.1) (NSTO) ceramics were fabricated using a conventional, high-temperature, solid state reaction technique. X-ray diffraction (XRD) patterns and Raman spectra confirmed that the tetragonal rutile is the main phase in all the ceramics. Additionally, XRD revealed the presence of a small amount of secondary phase of SiO_2_ in the co-doped ceramics. NSTO ceramics showed room temperature colossal relative permittivity (>10^4^) over the studied frequency range (1–1 MHz). (Nb, Si) co-doped (Nb_0.5_Si_0.5_)_0.025_Ti_0.975_O_2_ ceramics showed the most improved dielectric performance as higher ε′ (~5.5 × 10^4^) and lower tanδ (~0.18), at 10 kHz and 300 K, compared to pure TiO_2_ (1.1 × 10^3^, 0.34). The colossal permittivity of NSTO ceramics is attributed to an internal barrier layer capacitance (IBLC) effect, formed by insulating grain-boundaries and semiconductor grains in the ceramics. The electrical heterogeneity of the NSTO ceramics was confirmed by the impedance-spectroscopy analysis. The activation-energy values for conduction and relaxation of the co-doped NSTO ceramics are close to 0.1 eV which is reported for the dipolar relaxation due to electrons hopping between Ti^3+^ and Ti^4+^ ions in co-doped TiO_2_

## Figures and Tables

**Table 1:** Lattice parameters and volume cell for NSTO ceramics.

	Lattice Parameters (Å)		Cell Volume (Å^3^)
	a	c	
x = 0	4.59264	2.95668	62.3634
x = 0.025	4.59703	2.95852	62.5214
x = 0.05	4.59669	2.95655	62.4706
x = 0.01	4.60647	2.96170	62.8476

**Table 2:** The room temperature values of grain resistivity (R_g_), grain-boundary resistivity (R_gb_), ε′ and tanδ at 1.1 kHz and the minimum dielectric loss value (tanδ)_min_ for NSTO ceramics.

	R_g_ (Ω.cm)	R_gb_ (Ω.cm)	ε′	tanδ	(tanδ)_min_
			at 1.1 kHz		
x = 0	1.2 × 10^4^	9.0 × 10^6^	1.1 × 10^3^	0.65	0.34 (at 58 Hz)
x = 0.025	15.3	3.0 × 10^5^	5.5 × 10^4^	0.18	0.11 (at 10 KHz)
x = 0.05	9.7	1.0 × 10^5^	6.0 × 10^4^	0.40	0.11 (at 24 KHz)
x = 0.1	35.9	4.3 × 10^4^	5.3 × 10^4^	1.37	0.13 (at 47 KHz)

**Table 3:** Activation-energy values for conduction (E) and for relaxation process (U) for NSTO ceramics.

	E (eV)	U (eV)
x = 0	0.338	0.341
x = 0.025	0.089	0.095
x = 0.05	0.087	0.078
x = 0.1	0.101	0.099

## Data Availability

Data are available up on request.
